# Moderating effect of sleep quality on the association between hospital anxiety and quality of life in patients with mild to moderate dementia; A cross-sectional study

**DOI:** 10.1192/j.eurpsy.2023.2345

**Published:** 2023-07-19

**Authors:** K. Bosak, I. Filipčić, Ž. Bajić, V. Grošić

**Affiliations:** 1 Psychiatric Clinic Sveti Ivan, Zagreb; 2 Faculty of Dental Medicine and Health, Josip Juraj Strossmayer University of Osijek, Osijek; 3 School of Medicine, University of Zagreb, Zagreb, Croatia

## Abstract

**Introduction:**

Anxiety and sleep disorders are common and associated comorbidities of dementia. Previous studies has proven the association between anxiety and sleep disorder with a reduced quality of life in hospitalized patients with dementia. However, it is not clear whether the sleep disorders change the association between anxiety and quality of life.

**Objectives:**

To test the hypothesis that sleep quality modify the association between anxiety and quality of life in hospitalized patients with mild or moderate dementia.

**Methods:**

We performed this cross-sectional study during 2017 at University Psychiatric Hospital “Sveti Ivan”, Zagreb, Croatia. Data were collected on a consecutive sample of patients diagnosed with mild or moderate dementia. The outcome was the association between anxiety measured using the Hospital Anxiety and Depression Scale, and quality of life measured using the EQ-5D-5L visual-analogue scale. The independent variable was sleep quality measured using the Pittsburgh Sleep Quality Index (PSQI). We performed a moderation analysis using the Johnson-Neyman technique as implemented in Andrew F. Hayes macro “Process” Template 1, after adjusting for age, gender, education, body mass index, age at the time of onset of dementia, duration of hospitalization, severity of condition measured using Clinical Global Impression scale and Clinical Dementia Rating Scale, treatment with antidementives by multiple linear regression.

**Results:**

The median (interquartile range) age of 47 participants was 80 (76-83) years, 24 (51%) were women, 24 (51%) had nonspecific dementia, 16 (34%) Alzheimer’s Disease and 21 (46%) severe dementia. Hospital anxiety and quality of life were significantly, linearly, and inversely correlated both in bivariable, and in multivariable, adjusted analysis (r= -0.39; p=0.006; adjusted semipartial r= -0.41; p=0.017). After the adjustment for all covariates, the interaction between hospital anxiety, quality of life and sleep quality was not significant. However, hospital anxiety and quality of life were significantly correlated when PSQI score was ≥5.6, that is in 37% patients with the worst sleep quality.

**Conclusions:**

We partially confirmed the hypothesis that sleep quality modifies the association between hospital anxiety and quality of life in patients with mild to moderate dementia.

**Disclosure of Interest:**

None Declared

